# TECHNICAL BRIEF: Isolation of total DNA from postmortem human eye tissues and quality comparison between iris and retina

**Published:** 2012-12-22

**Authors:** Jay Ching Chieh Wang, Aikun Wang, Jiangyuan Gao, Sijia Cao, Idris Samad, Dean Zhang, Carol Ritland, Jing Z. Cui, Joanne Aiko Matsubara

**Affiliations:** 1 Ophthalmology and Visual Sciences University of British Columbia Vancouver, BC, Canada; 2 Department of Forest Sciences University of British Columbia Vancouver, BC, Canada

## Abstract

**Background:**

Recent genomic technologies have propelled our understanding of the mechanisms underlying complex eye diseases such as age-related macular degeneration (AMD). Genotyping postmortem eye tissues for known single nucleotide polymorphisms (SNPs) associated with AMD may prove valuable, especially when combined with information obtained through other methods such as immunohistochemistry, western blot, enzyme-linked immunosorbent assay (ELISA), and proteomics. Initially intending to genotype postmortem eye tissues for AMD-related SNPs, our group became interested in isolating and comparing the quality of DNA from the iris and retina of postmortem donor eyes. Since there is no previously published protocol in the literature on this topic, we present a protocol suitable for isolating high-quality DNA from postmortem eye tissues for genomic studies.

**Methods:**

DNA from 33 retinal samples and 35 iris samples was extracted using the phenol-chloroform-isoamyl method from postmortem donor eye tissues. The quantity of DNA was measured with a spectrophotometer while the quality was checked using gel electrophoresis. The DNA samples were then amplified with PCR for the *complement factor H (CFH)* gene. The purified amplified products were then genotyped for the SNPs in the *CFH* gene.

**Results:**

Regarding concentration, the retina yielded 936 ng/μl of DNA, while the iris yielded 78 ng/μl of DNA. Retinal DNA was also purer than iris DNA (260/280=1.78 vs. 1.46, respectively), and produced superior PCR results. Retinal tissue yielded significantly more DNA than the iris tissue per mg of sample (21.7 ng/μl/mg vs. 7.42 ng/μl/mg). Retinal DNA can be readily amplified with PCR, while iris DNA can also be amplified by adding bovine serum albumin. Overall, retinal tissues yielded DNA of superior quality, quantity, and suitability for genotyping and genomic studies.

**Conclusions:**

The protocol presented here provides a clear and reliable method for isolating total DNA from postmortem eye tissues. Retinal tissue provides DNA of excellent quantity and quality for genotyping and downstream genomic studies. However, DNA isolated from iris tissues, and treated with bovine serum albumin, may also be a valuable source of DNA for genotyping and genomic studies.

## Introduction

Since the discovery of the retinoblastoma gene in 1985, modern genetics and genomics have taken on a prominent role in the studies of many ocular diseases, such as age-related macular degeneration (AMD), photoreceptor degenerations, and others [1]. The Y402H single nucleotide polymorphism (SNP) in the *complement factor H (CFH)* gene, recently identified to be associated with increased risk for AMD, has become a prominent area of research [2-5]. Genetic studies, having already been shown to be valuable in understanding the etiology of several eye diseases [6-8] are, without a doubt, at the current forefront of ocular research. Furthermore, genetic information becomes even more valuable when combined with data obtained from other techniques, such as immunohistochemistry, enzyme-linked immunosorbent assay (ELISA), and proteomics.

Vision scientists who collaborate with eye banks and receive postmortem donor eye tissues are in a unique position to study the relationships between at-risk SNPs and disease phenotypes in human eyes. However, eye banks collect tissues primarily for using corneal tissues for transplantation, and secondarily for research. Under established guidelines, the Eye Banks of British Columbia, aside from eyes, collect only a small amount of blood for the sole purpose of identifying the suitability of the corneal tissue for transplantation.

Postmortem donor eye tissues hold valuable genetic information for studies of ocular disease; without accompanying additional tissues, investigators are faced with the task of isolating DNA directly from postmortem eyes. Thus, there is an urgent need for a method to isolate DNA from postmortem donor eyes. To our knowledge, this is the first study to present a protocol for isolating high-quality DNA from postmortem donor eye tissues to investigate the relationship between genetics and disease.

Previous studies have suggested that extracting DNA using commercially available DNA isolation kits may result in a lower yield of DNA compared to traditional methods [9-11]. It has also been the experience of our group that commercially available DNA isolation kits yielded DNA of lesser quality and storage potential. Hence, this protocol employs the phenol-chloroform-isoamyl (PCI) method of DNA extraction, which offers some major advantages. First, the method is less expensive than commercial kits in terms of the costs of supplies [12]. Second, the PCI method is especially suitable for isolating high-molecular-weight total DNA, as this method yields greater quantity than commercial kits [13]. Finally, DNA isolated under the PCI method can be stored at −20 °C indefinitely in the presence of ethanol for future studies [14].

This study also explored the quality and quantity of DNA isolated from different tissue compartments of the eye. After a pilot study on the concentration and purity of a few samples of DNA isolated from the optic nerve, retina, choroid, and iris, we narrowed the choices down to comparing DNA isolated from the retina and iris for suitability in genotyping studies.

## Material and Methods

### Postmortem donor eyes

All donor eyes were procured from the British Columbia Eye Bank. Ethics approval was obtained from the Clinical Research Ethics Board (CREB) at the University of British Columbia (UBC). All tissues were acquired with written, informed consent, in accordance with the principles outlined in the Declaration of Helsinki. All tissues in this study were obtained within 20 h from death of patients. Ages ranged from 34 to 79 years with a mean of 64 years.

In total, 33 retinal samples were used, while 35 iris samples were used. All samples were cut into 4 mm × 5 mm blocks for use.

### Reagents

1. Sodium chloride, tris(hydroxymethyl) aminomethane hydrochloride, EDTA (STE) buffer

2. 10% sodium dodecyl sulfate (SDS; Fisher Scientific, Ottawa, Canada, cat. BP1311). Cautions: Wear mask and avoid inhaling SDS powder during handling, as inhalation of SDS powder can cause microscopic damage to the lungs.

3. 100% ethanol (Leica, Concord, Canada, cat. 3,803,686)

4. 20 mg/ml proteinase K enzyme (Roche Applied Science, Indianapolis, IN; “Proteinase K, recombinant, PCR grade,” cat. 03,115,879,001). Add 0.02 g of proteinase K powder in 1 ml distilled water and aliquot to 200 μl volumes before storing at −20 °C.

5. 2% bleach (made from 5.25% bleach; Fisher Scientific, Ottawa, Canada, cat. 361,021,076). Caution: Be careful not to spill bleach on skin, in eyes, and in other mucus membranes. Avoid contact with clothing.

6. 5 M NaCl solution

7. 70% ethanol (made from 100% ethanol)

8. Chloroform/isoamyl alcohol (24:1; Fisher Scientific, Ottawa, Canada; “Isoamyl alcohol, Molecular biology grade,” cat. BP1150). Caution: Work in fume hood.

9. Phenol (pH=8.0, buffered; VWR, Edmonton, Canada; “Phenol, buffered, OminiPur. 99.0% min.,” cat. EM-6710). Caution: Corrosive; avoid contact with skin. Wear goggles and work in fume hood. If splash onto skin, let it run under cold water for 30 min. Do not heat phenol beyond 37 °C as it could explode at a higher temperature.

10. RNase A (powder; Roche Applied Science, Indianapolis, IN; “RNase A from bovine pancreas,” cat. 10,109,169,001). Mix together 10 mg of RNase A, 10 μl of 1 M Tris-HCl (pH 8.0), 3 μl of 5 M NaCl, and 987 μl of distilled water. Boil for 15 min and allow solution to slowly come to room temperature before use. Aliquot and store at −20 °C.

11. Albumin, bovine serum, Fraction V, approximately 99% (Sigma-Aldrich, Oakville, Ontario, Canada, cat. A3059).

All solutions are made with DNase/RNase free water for use in the experiment. When possible, solutions were autoclaved before use.

### Equipment

1. Centrifugal evaporator (Montreal Biotech Inc., UNIVAPO Vacuum Concentrators, Dorval, Canada)

2. Hybridization oven incubator (Boekel Scientific, Boekel Bambino II Mini Hybridization Oven, Feasterville, PA, cat. 230,301)

3. Spectrophotometer (NanoDrop 2000, Thermo Scientific, Ottawa, Canada)

4. 1.5 ml microtube (Fisher Scientific, “Fisherbrand Premium Microcentrifuge Tubes,” cat. 05–408–129)

5. Microtube rotator (VWR, Tube Rotator and Rotisseries, Edmonton, Canada, cat. 13,916-822)

6. Centrifuge (Thermo Scientific Sorvall Legend Micro 21R Centrifuge, cat. 75,002,446)

### DNA isolation

The time required for the method is 2–5 days. To avoid contamination, always wear gloves throughout all procedures, and sterilize all work surfaces including glove-covered hands with 2% bleach before and during extraction.

1. Cut each globe circumferentially at the pars plana to remove the anterior segment. Collect the vitreous, carefully dissect the retinal tissue, and remove from the retinal pigment epithelium and choroid. Cut the iris and the retina into 4 mm × 5 mm blocks for DNA extraction.

2. Place each tissue block (10–100 mg) into sterilized DNAase/RNase free 1.5 ml centrifuge tubes.

3. Add 360 μl of STE buffer, 40 μl of 10% SDS, and 4 μl of 20 mg/ml proteinase K to each tissue. Mix gently to resuspend tissue. Seal samples with parafilm.

4. Incubate samples at 55 °C in hybridization oven overnight.

5. Add 175 μl of buffered phenol and 175 μl of chloroform/isoamyl (24:1) to each sample. Mix the solution on a microtube rotator for 10 min and then centrifuge at 18.8x10^3^ ×g for 15 min.

6. Carefully pipette out as much aqueous phase as possible, leaving the interphase, and transfer to a new sterilized 1.5 ml microtube.

7. Add chloroform/isoamyl (24:1) into the supernatant at an amount equivalent to the supernatant obtained from the last step. Mix on the microtube rotator for 10 min. Then, centrifuge at 18.8x10^3^ ×g for 15 min.

8. Transfer as much supernatant as possible into new microtubes.

9. Add 10 μl RNase into each sample and incubate at 37 °C for 1 h.

10. Add chloroform/isoamyl (24:1) into the supernatant at an amount equivalent to the supernatant obtained from the last step. Mix on the microtube mixer for 10 min and centrifuge at 18.8x10^3^ ×g for 15 min.

11. Slowly pipette out 250 μl of each supernatant into a new 1.5 ml microtube and add 7.5 μl of 5 M NaCl into the supernatant to reach a final concentration of 0.15 M of NaCl in the sample and mix gently. Add twice the volume of supernatant of −20 °C, 100% ethanol. Mix samples gently by inversion. DNA will precipitate with a thread-like appearance.

12. If DNA precipitate is observed (i.e., strands of DNA are clearly visible), centrifuge at 18.8x10^3^ ×g for 10 min and carefully pipette out the supernatant, while retaining the DNA pellets. If DNA pellets are visible, leave the solution at −20 °C overnight, then centrifuge, discard supernatant, and retain the pellets.

13. Add 500 μl of −20 °C 70% ethanol into DNA pellets, invert the tube a few times, and then centrifuge for 10 min at 18.8x10^3^ ×g, 0 °C. Carefully pipette out the solution, saving the DNA pellets in the microtube.

14. Dry the DNA pellet with a SpeedVac (Thermo Scientific, Nepean, Ontario, Canada) for 10 min, or leave the microtubes open in a fume hood for about 15 to 20 min to allow residual 70% ethanol to evaporate. Resuspend the pellet in 75 μl of sterilized distilled water or Tris-EDTA (TE) buffer, Leave the pellet to resuspend at least two overnights at 4 °C or one overnight at room temperature in the fume hood.

15. Quantify the DNA concentration and purity with a spectrophotometer (Fisher Scientific, NanoDrop, model 2000C).

16. Perform agarose gel electrophoresis to check the quality of the DNA. Our laboratory has been using 1.2% gel with success.

17. To avoid repeated freezing and thawing of DNA, aliquot DNA in Eppendorf tubes per sample for long-term storage at −20 °C.

### PCR

#### Preparation of PCR master solution

dH_2_O 19.3 μl

dNTP (25 mM) 0.2 μl

Primer Mix (20 μM) 0.5 μl

10xPCR buffer (15 mM MgCL_2_) 2.5 μl

Taq DNA polymerase (5 U/μl) 0.5 μl

All PCR components above were multiplied by a factor of the number of genomic DNA being amplified. Mix the PCR master solution thoroughly. For each sample to be amplified, first add 2 μl of genomic DNA (25 ng/μl) into each corresponding well of the 96-well PCR plate (Applied Biosystems, cat. 4,346,906), then add 23 μl of PCR master solution, and mix the whole solution in the well by pipetting up and down several times. The PCR volume was 25 μl in total for each reaction. Seal the PCR plate with optical adhesive film (Applied Biosystems, Carlsbad, CA, cat. 4,311,971). All procedures were conducted on ice to prevent primers from non-specific annealing to template DNAs. Assemble the PCR plate onto the PCR machine (Applied Biosystems, 7500 Fast Real-Time PCR system). The PCR conditions were as follows: initial denaturing temperature of 94 °C (5 min), 39 repetitive cycles with denaturing temperature of 94 °C (30 s), annealing temperature of 57 °C (30 s), and extension temperature of 72 °C (60 s). The forward and reverse primers for PCR amplification of *CFH* rs1061170 [GeneBank accession: DQ233256] were 5′-AGT AAC TTT AGT TCG TCT TCA G-3′ and 5′-ATC TTC TTG GTG TGA GAT AAC G-3′, respectively, yielding a PCR product of 660 bp. Four μl of PCR product for each sample was run in 1.2% agarose gel at 70 V for 30 min. The gel pictures were taken by using a BioRad GelDoc imager (Mississauga, Ontario, Canada). In the PCR comparing the effect of BSA on reverting the inhibitory effect of melanin, 1 μg of BSA was added for each 1 μl of PCR solution.

### Purification of amplicons

To each corresponding well with 20 μl PCR product, add 100 μl of DNA binding buffer, pipette up and down 6X, and load the mixed PCR samples into PCR purification column (Qiagen, Toronto, Ontario, Canada, cat. 28,104). After brief centrifugation, wash the columns 2X by using wash buffer and finally obtain 40 μl purified PCR product in elution buffer according to Qiagen’s PCR purification protocol.

### Genotyping

The concentration of purified PCR products was determined by using a NanoDrop (Ottawa, Ontario, Canada) spectrophotometer. The purified PCR products were mixed with sequencing primer at suitable concentrations as required and then sent to GENEWIZ Inc. for sequencing analysis. The *CFH* sequencing primer was 5′-ACT TTA GTT CGT CTT CAG-3′.

### Statistics

All statistical analyses were performed using the Student *t* test, with the threshold value set as p<0.05.

## Results

**Figure 1:**
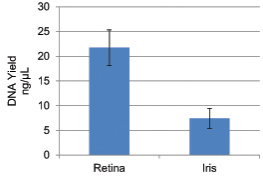
Average concentration of DNA yielded by each mg of tissue used. On average, 21.7 ng/μl (standard error=3.63 ng/μl) of DNA was yielded per 1 mg of retinal tissue used. As for the iris, on average, 7.42 ng/μl (standard error=2.01 ng/μl) of DNA was yielded per 1 mg of iris tissue used. The difference is statistically significant (p<0.01).

We compared various characteristics of DNA isolated from the iris and retina, and found that the quantity and quality of DNA obtained from the retina are significantly superior to that of the iris when using the same starting amount. After the same volume of dH_2_O (75 μl) was added to the DNA pellets, the average concentrations of DNA obtained from the retina and iris were 936 ng/μl (max.=2900.8 ng/μl, min. 189.6 ng/μl, SD=645 ng/μl) and 78 ng/μl (max.=379.6 ng/μl, min.=3.1 ng/μl, SD=78 ng/μl), respectively (p<0.01). In addition, the retina also yielded significantly purer DNA than the iris (260/280_retina_=1.78, max. 260/280_retina_=1.86, min 260/280_retina_=1.52, SD=0.07 VS 260/280_iris_=1.46, max. 260/280_iris_=1.75, min. 260/280_iris_=0.69, SD=0.18 respectively; p<0.01), suggesting that retinal DNA has less protein contamination. On average, retinal tissues yielded 21.7 ng/μl of DNA per 1 mg of tissue used, while iris tissues yielded 7.42 ng/μl of DNA per 1 mg of tissue used (p<0.01; Figure 1).

**Figure 2:**
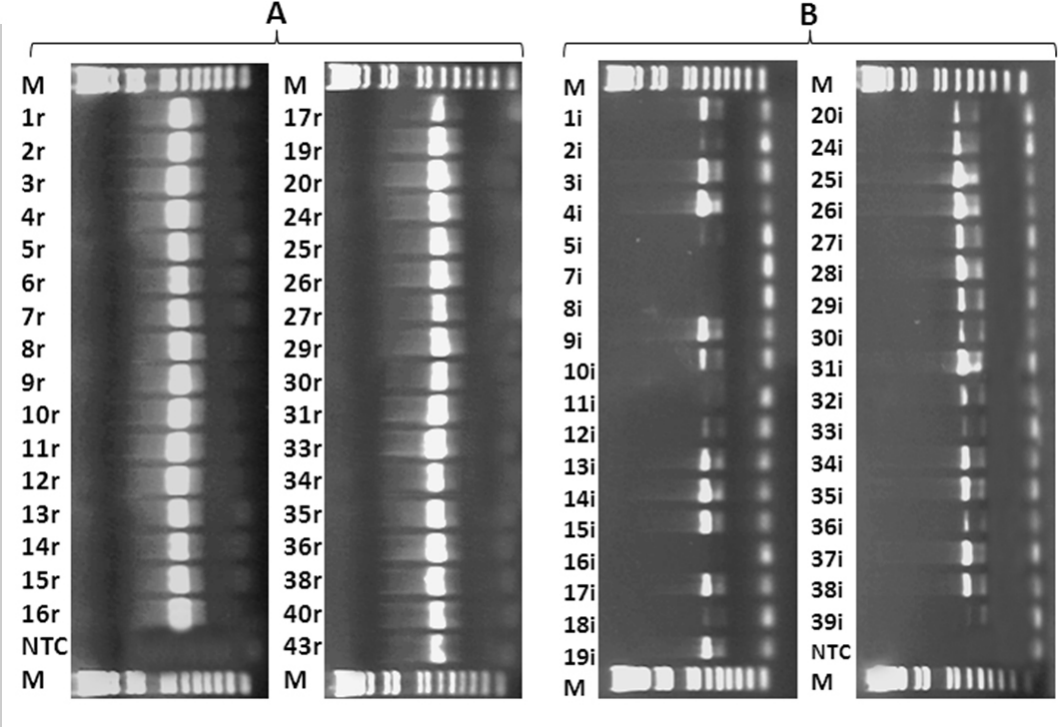
Results of 1.2% agarose gel electrophoresis for amplified *CFH* products. Each lane represents an individual DNA sample. The number assigned to each lane corresponded to the arbitrary labels given to each donor. “R” represents retinal DNA while “I” represents iris DNA. Thus, 1r and 1i represent retinal and iris DNA, respectively, obtained from donor eye number 1. For each sample, 4 μl of PCR product (out of 25 μl) was used and ran at 70 V for 30 min in 1% agarose gel. **A**: Every retinal DNA sample yielded detectable PCR product. **B**: The PCR products from the iris DNA samples were less prominently detected than retinal DNA. Some samples did not yield detectable products. Retinal DNA bands are significantly brighter than the iris DNA bands, suggesting greater yields. Note: M=Marker (1 kb DNA ladder, Invitrogen); NTC=non-template control (negative control).

**Figure 3:**
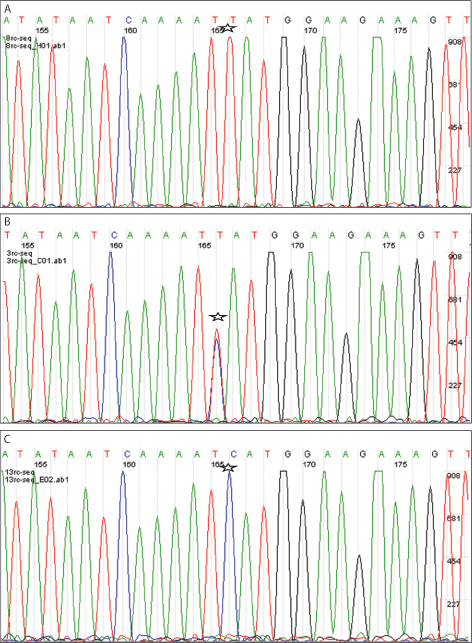
Shown here are samples of *CFH* sequencing results from three representative retinal DNA samples. Position 166 indicates the location of the *CFH* Y402H single nucleotide polymorphism (SNP). **A**: The SNP position (166 bp) indicated with a star exhibited a wild-type TT allele. **B**: The SNP position (16 6bp) indicated with a star exhibited a heterozygous TC allele. **C**: The SNP position (166bp) indicated with a star exhibited a homozygous CC allele. These sample results showed that the proposed method isolated DNA of quality sufficient for genotyping.

To confirm the suitability of the DNA obtained from the iris and retina for future genotyping studies, we amplified a fragment of the *CFH* gene, including the Y402H SNP, via PCR [15]. DNA gel electrophoresis of the PCR products showed that retinal DNA yielded superior PCR products. Every retinal DNA sample yielded prominent PCR products, while the PCR products of the iris DNA generally gave lower or no detectable yield (Figure 2). Under visual inspection, the retinal DNA bands were brighter in intensity. This further suggested that the retina yielded DNA more suitable for PCR amplification and further genotyping and genomic studies than the iris. Subsequently, the *CFH* gene fragment amplified from retinal DNA was successfully sequenced via Sanger sequencing (Figure 3).

**Figure 4:**
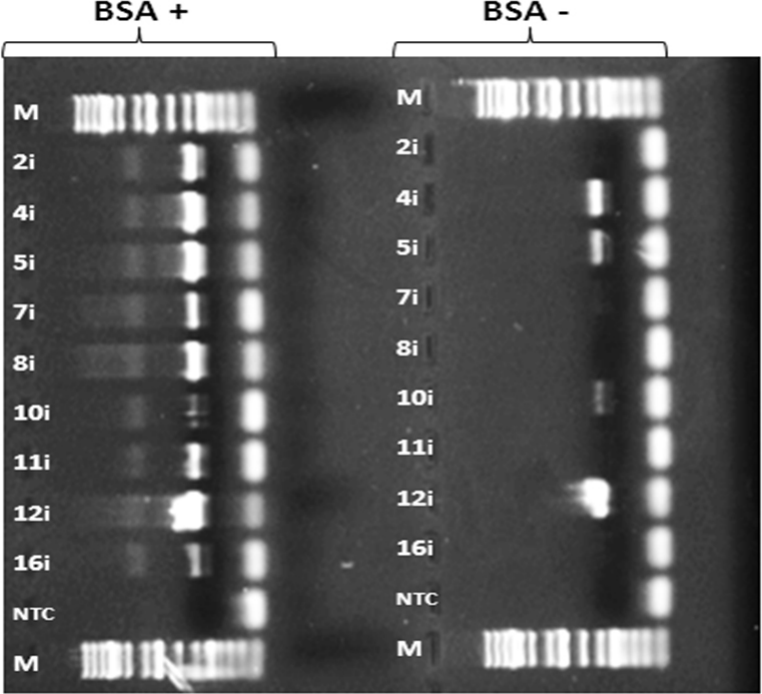
BSA fraction V significantly improved polymerase chain reaction (PCR) yield in iris DNA. A set of parallel PCR was performed to compare the effect of BSA on PCR amplification of DNA obtained from iris tissues. The PCR conditions used in both groups were the same as that described in the method section and Figure 2, with the exception that the BSA+ group contained 1 μg of BSA per 1 μl of PCR solution. Each lane represented an individual iris DNA sample, with the number an arbitrary label assigned to each donor. The “i” following each number indicates that the DNA is of iris origin. Note: M=Marker (1 kb DNA ladder, Invitrogen); NTC=non-template control (negative control).

Although retinal tissues yielded DNA more suitable for PCR amplification than iris tissue, the PCR amplification of iris DNA can be improved. To test the hypothesis that melanin inhibits PCR amplification of iris DNA, parallel PCRs were run, with and without bovine serum albumin (BSA), as previous studies have suggested that BSA suppresses the inhibition of PCR by melanin [15]. The results showed that iris DNA with BSA treatment yielded much better PCR results than the non-BSA-treated samples, indicating that treatment with BSA improved PCR amplification of iris DNA (Figure 4).

## Discussion

### Why use postmortem eyes for isolating DNA?

Under appropriate consent, eye banks enucleate eyes from postmortem donors for corneal transplant purposes, and forward the remaining eye tissues to laboratories for scientific research. According to established guidelines, eye banks may collect a small amount of blood and splenic samples, which are reserved solely for determining transplant suitability, such as infectious disease status. Thus, many researchers are limited to using eye tissues alone to study relationships between genotype and phenotype. This situation is similar to the need to isolate DNA from formalin fixed paraffin embedded tissues, which, like postmortem donor eyes, hold valuable genetic information [16,17]. However, studies using archival formalin fixed paraffin embedded eye tissues may also encounter the lack of readily available tissues from the original donor for genotyping purposes. Our study addresses this issue by providing investigators a method for isolating DNA from eye tissue, while still preserving the bulk of eye tissue for other analyses and correlative studies.

### Quantity and quality of DNA isolated from the retina compared to the iris

Our results showed that retinal tissue yielded more DNA, compared to iris tissue. Furthermore, retinal DNA yielded more robust results when amplifying for the *CFH* gene with PCR. With higher DNA yield per unit of mass, and the ability to be readily amplified by PCR, retinal tissue seems to be a promising tissue candidate for extracting DNA for genetic studies.

### Possible explanation for the higher yield per unit mass of retinal DNA compared to iris DNA

The retina might yield genomic DNA of superior quality and quantity because the retina is more densely packed with cells than the iris. On histologic sections, three cell-rich layers packed with nuclei can be clearly seen [18]. However, the iris is composed largely of stroma (including fibroblasts and melanocytes loosely scattered throughout), constrictor papillae made of smooth muscles, and two layers of cuboidal epithelium [18]. Under histologic evaluation, iris tissue exhibits less cellular density than retinal tissue [18]. Thus, the retina may yield more DNA per unit mass due to the higher cellular density.

### Inhibition of polymerase chain reaction amplification on iris DNA by melanin and effect of bovine serum albumin on minimizing the inhibition

Our data showed that the result of PCR amplification on iris DNA is less optimal than that of retinal DNA (Figure 2). Previous studies suggested that melanin, a product of melanocytes found in the iris, is a potent inhibitor of PCR [19]. In particular, melanin was suggested to interact with the thermostable DNA polymerase used in PCR, thus reducing the ability of the polymerase to synthesize new DNA strands [19]. Previous studies also suggested that BSA may reverse melanin-induced PCR inhibitions [15,19]. Therefore, a set of parallel PCRs was performed to compare the effect of BSA on amplifying a few randomly selected iris DNA samples obtained from donor eyes. Our results suggested that melanin participated in inhibiting PCR in the iris DNA, and that BSA treatment significantly improved the amplification of iris DNA (Figure 4). Thus, perhaps the iris, with appropriate BSA treatment, could be used for genotyping purposes, leaving all retinal tissues available for other analyses.

### A suggested approach for using postmortem eye tissues obtained from eye banks

Postmortem eye tissues, since they are often forwarded to laboratories in pairs, offer a unique opportunity to study the relationship between genotype and disease pathogenesis and manifestations. The results of this study suggest that when only postmortem eye tissues are available for genetic studies, retinal tissue offers a readily available source of DNA. Investigators can obtain DNA from the retina of one eye while preserving the other eye for protein studies such as immunohistochemistry, enzyme-linked immunosorbent assay, western blot, and proteomics, yielding valuable correlative data to understand the relationship between genotype and phenotype in ocular diseases. If the retinal tissues of both eyes are required for analysis, investigators can obtain DNA from iris tissue, by adding BSA to enhance DNA amplification for genetic studies.

### Conclusion

The protocol presented here is suitable for isolating total DNA from postmortem eye tissues for genotyping and genomic studies. To obtain the best quality and quantity of DNA, we recommend isolating total DNA from retinal tissues. Iris tissues can be used if retinal tissue is not available, or if retinal tissues must be preserved for other study purposes, by adding BSA in the PCR solution to enhance amplification.
